# Representations of long-acting antipsychotics in patients at the Arrazi hospital in Salé Morocco

**DOI:** 10.1192/j.eurpsy.2023.603

**Published:** 2023-07-19

**Authors:** H. Chebli, A. Tounsi, H. Berrada, F. Laboudi, A. Ouanass

**Affiliations:** ^1^University Psychiatric Hospital Arrazi in Sale, Sale; ^2^University Psychiatric Hospital Arrazi in Sale, Casablanca, Morocco

## Abstract

**Introduction:**

Since the appearance of long-acting antipsychotics (LAPAs) and given the high frequency of non-adherence to treatment in psychotic disorders, LAPAs have recognized a resurgence of interest in the psychiatric literature. These long-acting drugs may pose ethical issues (e.g. limitation of freedom).

**Objectives:**

The present study aims to determine the representations of long-acting antipsychotics in patients followed at Arrazi Hospital in Salé.

**Methods:**

Descriptive study carried out with patients hospitalized at the Arrazi hospital in Salé and those followed in consultation who are on APAP or who have already used it. The collection of information is done using an exploitation sheet

**Results:**

APAPs have been used for less than 5 years by 53.8% of patients84.6% of participants do not use APAP bychoice, in 79.2% of cases it was the doctor’s decision and in 20.8% of cases it was the family’s choiceMonotherapytreatment was the most cited benefit by our patients (76.9%)The route of administration of APAP by intramuscular injection is the problem encountered in 57.7% of our patients, while11.5% of patients find no inconvenience for the use of these psychotropics.

**Conclusions:**

Negative beliefs associated with the treatment contribute to a very large part to the lack of compliance, onthe contrary, long-acting antipsychotics may be better accepted by patients when taking into account the patients’ beliefsand preferences in the development of the treatment. therapeutic project

**Disclosure of Interest:**

None Declared

